# The Effects of Reproductive Disorders, Parity, and Litter Size on Milk Yield of Serrana Goats

**DOI:** 10.3390/ani9110968

**Published:** 2019-11-13

**Authors:** Gisele Margatho, Vicente Rodríguez-Estévez, Hélder Quintas, João Simões

**Affiliations:** 1 Animal and Veterinary Research Centre (CECAV), University of Trás-os-Montes e Alto Douro, Quinta de Prados, 5370-801 Vila Real, Portugal; giselemargatho@gmail.com; 2 Department of Animal Production, University of Córdoba, Campus de Rabanales, 14071 Córdoba, Spain; vrestevez@uco.es; 3 Mountain Research Centre (CIMO), School of Agriculture, Polytechnic Institute of Bragança (IPB), Campus de Santa Apolónia, 5300-253 Bragança, Portugal; helder5tas@ipb.pt

**Keywords:** abortion, benchmarking, goats, kidding, pregnancy, prolificacy, milk production

## Abstract

**Simple Summary:**

This study aimed to evaluate the effects of reproductive disorders, parity, and litter size on a 150-day standardized milk yield of the Transmontano ecotype of the Serrana goat breed (a dual purpose breed) over a three decade period. The 150-day standardized milk yield was significantly influenced by all studied factors and their interactions. The milk yield reduction due to reproductive disorders was more intense in primiparous than multiparous goats, and in primiparous or multiparous goats of the Transmontano ecotype presenting multiple fetuses, than in those presenting singletons. This milk production pattern proves that the lactation following abortion is viable for production purposes and an improvement of production system management can play an important role in mitigating milk yield losses.

**Abstract:**

Several reproductive factors may affect milk yield in goats. The main aim of this study was to evaluate the influence of reproductive disorders, parity, and litter size, and their interactions on the 150-day standardized milk yield (SMY150) of low-producing dairy goats extensively raised. A total of 148,084 lactations between 1993 and 2015 were obtained from data of the Genpro pedigree records of the Transmontano ecotype of Serrana goat breed. The presence or absence of reproductive disorders (RD) from late (>half) pregnancy (abortions followed by lactation) or at kidding, number of fetuses (single vs. multiple), and parity (primiparous vs. multiparous) of the Transmontano ecotype of Serrana goat were used as fixed effects to fit a general linear model for a SMY150 output. A significant effect (*p* < 0.001) of all factors on SMY150, as well as three-way interactions, were observed. The SMY150 reduction subsequent to RD was 3.7% for multiparous and 9.6% for primiparous goats carrying singletons, and 14.1% for multiparous and 18.8% primiparous goats carrying multiple fetuses. It was concluded that a new lactation following abortion occurrence is viable for production purpose in low-producing dairy goats under pastoralism. Nevertheless, the impact of RD on SMY150 varied according to the number of fetuses and the parity of the Transmontano ecotype of Serrana goats. This information should be used in decision-making practices regarding reproductive and herd health management.

## 1. Introduction

Livestock benchmarking calculates data surrounding production, the resource base, costs, and other factors associated with the farming business to deduce effects from farm-recorded data. This is a useful and common management technique for livestock systems [1,2]. However, inferring causal effects between variables from farm data is challenging, as the association between them may arise not only from the effect of one on another but also from confounding background factors [3].

In dairy production, many factors may affect the overall milk yield (MY) and composition [4,5]. It is crucial to understand which factors directly affect MY and quality, such as diet [6], management [7], udder health, and somatic cell counts [8]. The influence of the number of fetuses or offspring and parity on MY is not straightforward in ruminants, and somewhat controversial in dairy versus meat goats. Ewes giving birth to more than one lamb produce more milk per lactation [3]; and those nursing twins produce more milk than those nursing single lambs [9,10]. 

There are different animal-dependent factors which affect goat prolificacy [11]. Morantes et al. [12] indicated a significantly higher prolificacy for dairy goat farms using assisted reproductive techniques compared to unassisted farms. In goats, multiple births have a higher mean MY in spite of having longer lactations than single births [13,14,15]. Thomas et al. [16] showed increases of 27% in the MY of goats producing twins over those producing single kids and of 16% in goats producing triplets over those with twins, with no indication that this trend was affected by age or lactation number. However, Carnicella et al. [15] observed that goats with higher parities, in particular goats in greater than or equal to the fourth parity, had a longer lactation and consequently a higher total MY; in addition, goats kidding twins yielded more milk and had longer lactation.

Nevertheless, the influence of reproductive disorders (RD) such as abortion, parity, and litter size on MY, especially focused in 150-day standardized milk yield (SMY150), and their interactions, have not yet been fully studied in low-producing dairy goats with dual purpose (meat and milk). Increasing kid production is of great interest to goat farmers, especially in traditional systems; and thus, prolificacy is an economically important trait and the most important reproductive parameter in determining the efficiency of the system and the economic viability of the flock [17].

Recently, Simões and Pires [18] observed that the SMY150 in low-producing goats was negatively influenced by RD in late pregnancy (abortions) or at kidding (stillbirths and other perinatal mortality). In that same study, preliminary data showed a higher frequency of RD in primiparous versus multiparous goats, and in single pregnancies versus pregnancies with multiple fetuses. Therefore, a more thorough evaluation also involving the number of fetuses is needed to address the best goat production management. Management and decision-making practices that aim to increase prolificacy could positively affect MY. This would be very useful knowledge at the farm level, especially for traditional grazing systems where MY is key to food production and economic security of farmers.

This is a retrospective study aiming to determine the relationship between RD, parity, and litter size on the SMY150 of the ecotype Transmontano of Serrana goats, a Portuguese local breed reared in extensive systems. The ultimate goal is to improve benchmarking knowledge in these low-producing dairy goats.

## 2. Materials and Methods

### 2.1. Animals and Records

A total of 148,084 lactations were obtained, between 1993 and 2015, from data of the pedigree records of Serrana goats breed (Genpro, Ruralbit, Portugal). For a description of this breed see Sociedade Portuguesa de Ovinotecnia e Caprinotecnia [19]. The Transmontano ecotype of this breed spread over northern Portugal and was considered for this study. 

These regions are included in the temperate Mediterranean climate region, classified as Csa (dry and hot-summer climate) or Csb (dry and warm-summer climate) according to the Köppen climate classification [20]; Overall, and considering the data reported between 1971 and 2000, accessible at [21], the annual rainfall varies from 144.1 mm per month in winter (December) reaching a tenfold decrease, 13.7 mm, in summer (August). Inversely, the minimum, maximum, and average temperature is low in January (4.5, 13.1, and 8.8 °C, respectively), and increases gradually reaching the highest values in August (15.5, 28.8, and 22.2 °C, respectively), followed by a gradual decrease until the next winter. In mountain zones, these temperatures have a much wider range. Most recent climatic data can be found at the Portuguese Institute for Sea and Atmosphere site [22].

The production system was described by Simões and Bauer [23] and by Margatho et al. [24]. Briefly, these goats were reared under daytime pastoralism, mainly in mountain regions, in herds normally up to 100 adult animals. The feeding varies according to the seasonal biomass and goats can move several kilometers per day during grazing, mainly in mountain zones. The natural potential vegetation from both regions was included in a spatial model reported by Capelo et al. [25]. Nevertheless, some forages (e.g., rye grass and sorghum) are cultivated in few herds. The stocking rate is estimated 0.2–0.3 goats per hectare. Normally, rye grass and salt are fully available in stable areas. A concentrated feed, normally between 0.25 and 0.75 kg, is individually supplemented to each lactating female at milking time, which is normally performed by hand once a day. The prevalent practice is milking goats after a sucking period up to 75 days. Natural mating, keeping bucks together with does, is the common practice, without artificial insemination, and genetic improvement remains incipient. The fertility rate can reach 95% in herds and the first kidding occurs approximately at 15 months of age. In the reproductive season, some goats are simultaneously pregnant and lactating. The annual replacement rate varied approximately by 20% throughout the period studied. Sanitary measures include de-worming and vaccination programs, such as enterotoxaemia, as well as against brucellosis in some cases and in some areas. Similar to other worldwide regions [26], milking goats after abortion is a recurrent practice.

For the present study, each record included ecotype, number of fetuses, parity (1 to ≥14) or lactation number, month of kidding or abortion, RD data as independent variables, and SMY150 as a dependent variable. The RD mainly included abortion from late (>half) pregnancy with a subsequent new lactation. Abortion was defined as the delivery of one dead fetus or fetuses before day 140 of pregnancy. However, throughout the three decades studied, due to the practical difficulty in classifying the exact time of mating, RD occurring in the last two weeks of pregnancy, such as stillbirth occurrence, mummified fetus, and other perinatal mortality were also considered. Stillbirths were defined as premature or at term fetuses (≥140 days) born dead or dead within 24 h after birth, while abortions are early or late pregnancy dead fetuses without possibilities of survival. Mummified fetus was defined as (aseptic) dead fetus with reabsorption of body and fetal fluids. These abnormalities were registered by farmers and supervised by technicians during their periodical interventions in herds and transferred to the Genpro database. 

The data, milk measurements, and milk samples (for milk composition evaluation) regarding one-day milk production were collected monthly from the ≤52nd (goats without suckling period) or ≤97th (after suckling period) day on lactation to the end of lactation [27]. Each lactation was only considered for at least three and four successive evaluations in primiparous and multiparous lactating goats, respectively. The Fleischmann’s method [28] was used to estimate the 150-day standardized milk yield (SMY150). 

### 2.2. Statistical Analysis 

The means of SMY150 by parity/lactation number were tested by ANOVA and the Tukey test.

Univariable logistic regression models were made to test the effects between each parity or number of fetuses and RD occurrence. The Wald test was also used a posteriori and the odd ratios calculated. 

An analysis of mean for proportions was made for the RD estimation according to the month in order to determine which proportions are significantly different from the overall average. For this purpose, the likelihood ratio test was used and upper and low decision lines (α = 0.05) were estimated according to Nelson et al. [29].

To build the general linear model, parity was classified as primiparous or multiparous goat, the number of fetuses/kids as single or multiple (two or more) and the RD as presence or absence. 

The three fixed effects and their interactions were fitted by the following equation:

Y_ijkl_= u + F_i_ + P_j_ + R_k_ + (FP)_ij_ + (FR)_ik_ + (PR)_jk_ + (FPR)_ijk_ + e_ijkl_ (1)

where

Y^ijkl^ = 150-day standardized milk production, (2)

u = Overall mean,(3)

F_i_ = fixed effect due to ith number of fetuses /kids (i = 1, 2), (4)

P_j_= fixed effect due to j^th^ parity (j = 1, 2), (5)

R_k_ = fixed effect due to k^th^ RD (k = 1, 2), (6)

(FP)_ij_, (FR)_ik_ and (PR)_jk_ = two-way interactions between F, P and R factors, (7)

(FPR)_ijk_ = three-way interactions between F, P and R factors, (8)

e_ijkl_ = random error. (9)

The Tukey–Kramer test was used to compare differences between pairs.

The JMP11 program from SAS [30] was used for all evaluations, and *p* < 0.05 was considered significant.

The results are show used a posteriori as mean ± standard deviation (±SD).

## 3. Results

### 3.1. Milk Yield, Prolificacy, and Reproductive Disorder Incidence 

The mean SMY150 observed was 96.6 ± 39.8 L (mean ± SD) with a 95% confidence interval (95% CI) between 96.4 and 96.8 L (*n* = 148,084). The mean SMY150 was 90.1 ± 32.2 L (*n* = 35,283) at the first lactation and reached a maximum of 100.2 ± 40.7 L (*n* = 22,362; *p* < 0.001) at the third lactation, decreasing gradually in the following lactations. However, SMY150 remains higher than the first lactation until to the 10th lactation (93.8 ± 39.4 L; *n* = 2317).

The prolificacy was 1.43 ± 0.55 (95% CI: 1.43 to 1.44). The frequency of singleton, twins and triplets was 58.9% (*n* = 87,229), 38.5% (*n* = 57,018), and 2.6% (*n* = 3837), respectively.

The overall incidence of RD was 9.4% (*n =* 13,882; 95% CI: 9.2 to 9.5) of the lactations. The RD were approximately twice (odds ratio = 1.87; 95% CI: 1.81 to 1.94) as likely to occur in primiparous (13.9%; *n =* 4914) than in multiparous goats (8.0%; *n =* 8968; *p* < 0.001). The incidence of RD also is higher in goats carrying singletons (15.0%; *n =* 13,056) than goats carrying twins (4.6%; *n =* 636) or triplets (5.0%; *n =* 190).

The incidence of RD also varied according to month (*p* < 0.001). September (6.3%; *n =* 7845), October (4.4%; *n =* 17,178), November (6.8%; *n =* 10,494) and December (8.1%; *n =* 15,977) were outside the lower decision line. Inversely, February (12.4%; *n =* 19,441), March (11.4%; *n =* 23,161), April (11.3%; *n =* 15,544), May (10.8%; *n =* 6686), June (12.6%; *n =* 2738), and July (12.1%; *n =* 1585) were outside the respective upper decision lines.

### 3.2. Effects of Litter Size, Parity, and Reproductive Disorders on Milk Yield

The general linear model reported significant effects (*p* < 0.001) of RD, number of fetuses, parity and their interactions on SMY150, as shown in Table 1.

Significant SMY150 differences (*p* < 0.001) were more pronounced in primiparous than multiparous goats and in goats carrying singletons than multiple fetuses (Table 2). 

## 4. Discussion

### 4.1. Milk Yield

Similar to previous studies in ruminant species a higher total MY was achieved in Transmontano ecotype of Serrana goats with multiple parity (*p* < 0.001). Parity plays an important role affecting milk production. In the present study, an increase of SMY150 was observed from the first to the third lactation, and persisted higher than the first lactation until the 10th lactation. An increasing MY as parity increased from first to third was also described by Salama et al. [31] for the Murciano-Granadina breed and Ciappesoni et al. [5] for Czech White Shorthaired breed. However, although most goat breeds reach maximum MY at the third or, less frequently, at the fourth lactation, Crepaldi et al. [32] found that MY showed a steady growing trend from first to fifth lactation in Alpine goats. Exceptionally, Vecerova and Krizek [33] found increasing MY until the 10th lactation in Czech White Short-hair goats. Carnicella et al. [15] observed that animals with higher parities, in particular goats of greater than or equal to the fourth parity, had a longer lactation and consequently a higher MY. In a recent study involving more than 300,000 lactations, Arnal et al. [34] observed a significant and progressive decrease in MY after the fourth lactation in French goat breeds.

In other species, such as sheep, the effect of parity on MY is controversial. Different authors observed significant increases on MY as the number of lactations advanced [35,36], while others observed that parity had no significant effect on MY [37,38,39]. Inconsistent findings may be due to factors such as the number of lambs suckled, which can play a role when different parities are compared [38].

### 4.2. Prolificacy 

The average prolificacy of 1.43 we observed in Transmontano ecotype of Serrana goats is similar to that observed in other breeds such as 1.41 in Nubian breed in India [40], 1.45 in Alpine breed in Egypt [41], 1.49 in crossbred dairy goats of the Brazilian northeastern [42] and 1.51 in Florida breed in Spain [12]. Dickson-Urdaneta et al. [43] found a close prolificacy in Nubian does, with 1.38 kids. According to Sacoto and Simões [44] prolificacy of the Serrana goats (*p* < 0.001) depends on parity, reaching its maximum around the 5th kidding. Other authors reported that prolificacy is affected by the year of kidding [43,45]. Prolificacy of Portuguese Serrana goats has been shown to be influenced by the month of kidding, decreasing from December onwards and until a minimum in April [44].

### 4.3. Incidence of Reproductive Disorders

The proportion of RD (9.4%) found in this study is higher than the values previously reported by other authors, even more so considering that only the failures that precede lactations are included. In this regard, Menzies [46] and Braun [47] suggested 5% as a critical level of incidence of abortion and dystocia. Simões and Pires [18] noticed the risk for misclassification of abortion, especially for stillbirths due to dystocia at kidding time. A higher incidence of RD in first parity females has been reported in most herds and also in small ruminant species [48,49].

The occurrence of abortions also appears to be influenced by parity. Mellado et al. [50] showed that goats of different breeds in Mexico of parity groups two to five were half as likely to abort as primiparous or older goats. In that study, the oldest does were 90% more likely to have stillbirths than the younger goats, and the risk of stillbirth was lowest for goats of the fifth parity or less [50].

In the present study, primiparous goats had approximately twice the chance of presenting RD than all other parities. This fact could be justified, at least partly, by abortions caused by the presence of brucellosis during these three studied decades [51,52,53] and dystocia, which are more frequent in primiparous goats [54,55,56]. Currently, brucellosis can be considered eradicated in Portuguese goat farms and only few numbers of seropositive animals to *Brucella* can be detected in circumscribed zones [57]. Other infectious diseases, such as chlamydiosis, campylobacteriosis, Q fever, toxoplasmosis and leptospirosis, and even non-infectious causes of abortions probably played an important role in RD occurrence and should be monitored at the farm level [54].

In the present study, the proportion of RD varied seasonally, with the lowest proportions observed between September and November (4.4% to 6.8%). During fall, only a low number of goats are pregnant due to the natural breeding seasonality. This probably explains the relative low proportion of RD during this period. Inversely, in late winter, the great concentration of goats presenting advanced pregnancy and the high number of parturitions favor a higher circulation of infectious agents potentially causing abnormal parturitions which increase RD. Heat stress during hot months between spring and summer, can also influence the occurrence of RD. For example, in a hot arid environment in Mexico, Mellado et al. [50] described a low frequency of stillbirths in goats bred in the fall. In Spain, Morantes et al. [12] also observed an influence of month on abortion in Florida breed goats from 51 farms in 2010, and where about 75% of farmers used hormonal protocols to change the reproductive seasonality. Still, the lowest frequency for the whole set of abortions (<2.5%) was observed from May to July. These findings indicate the possible influence of other infectious and non-infectious factors such as heat stress on the occurrence of abortion in extensive production systems. 

### 4.4. Effect of Reproductive Disorders, Parity, and Number of Fetuses on Milk Yield and Their Interactions

In the present study, we observed significant effects of RD, parity and No. of fetuses on SMY150 in Tansmontano ecotype of Serrana goats. All groups consistently displayed a decrease in SMY150 due to RD, but at different degree due to interactions between the studied factors. In a previous study, Simões and Pires [18] also observed a significant negative effect of RD on the subsequent lactations in both, primiparous and multiparous, goats of the Transmontano ecotype. Further, they observed similar values of SMY150 in primiparous goats carrying either single or multiple fetuses in presence of RD. This suggested the presence of interactions, which were included as a whole model in the present study.

In the present study, the overall effect of the number of fetuses on SMY150 is in agreement with other reported findings. For example, in Murciano-Granadina dairy goats [58] the MY increased up to the lactation peak for more prolific goats; and in Zaraibi goats [59] the litter size was positively related with an increase in MY in early lactation. In fact, MY is proportional to the mammary alveolar surface area which is positively correlated with fetal number. This relation may be due to the high levels of the hormonal placental lactogen produced by fetuses during the final phase of gestation [13,60]. Placental lactogen has a significant role in the control of normal mammary development and function in dairy goats. It provides prolactin hormone-like and growth hormone-like activities required for udder growth [60]. Multiple gestations are associated with more placental tissue and, hence, with elevated placental lactogen, suggesting that the extent of mammogenesis depends in part on the number of feto-placental units or placental mass [16]. Hayden el al. [13] emphasize the influence of the placental mass on the growth of mammary gland by the fact that in hand-milked goats, MY was related to the number of kids born by goat, implying that the kids in utero actively affect their chance of survival by regulating their food supply. We think that hormonal and mammary gland differences between primiparous and multiparous could partly explain the variation of SMY150 in the presence of RD.

Ferreira et al. [3] also observed a positive influence of prolificacy on MY in dairy sheep. Lactating ewes lambing multiple kids produced 12.62 L of milk during whole lactation more than ewes lambing singletons. Moreover, to reinforce the influence of prolificacy on MY, Delgado-Pertíñez et al. [61] found greater MY in the first to fifth weeks of lactation with two kids vs. one regardless of type of milking, i.e., machine-milking or natural suckling. Contrarily, MY did not differ during the post-weaning period (after the sixth week). Equally, Crepaldi et al. [32] found less effect of prolificacy on MY in late lactation. Peris et al. [62] showed the effect of prolificacy on udder volume, goats with twins having the largest volume and this was positively correlated with MY (*r* = 0.69). Therefore, it is likely that the occurrence of a fetal death, or abortion, would have a major impact on post-partum lactational performance, since it is related to pre-partum mammary gland development. 

It is well-known in goats, that multiple births have higher (total) mean MY in consequence of longer lactations [5,13−15,61]. In hand-milked goats and after correction for lactation number, Hayden et al. [13] observed that dams kidding twins and triplets had an increase of 27% and 47% MY, respectively, than dams kidding singletons. In Alpine goats, Crepaldi et al. [32], found that the difference in MY due to prolificacy was greater for first compared to third parity dams kidding twin kids than single kids (i.e., 63.2 vs. 15.02 kg of milk). Likewise, in sheep, the number of lambs is also a major factor influencing MY. Ewes nursing twins produce more milk than those nursing single lambs [9,10].

In the present study, the effect of the number of fetuses was observed considering SMY150. The standardization of lactation, and its evaluation instead of total MY, is very important due to the implications of the reproductive management to shorten the interval between kidding in seasonal species, such as small ruminants. Dairy goat farms using any reproductive technique obtain a significant higher prolificacy [12,63] hence, these techniques should be considered as an indirect method to achieve a higher total MY and SMY150.

Suckling induces prolactin secretion which is essential for maintaining lactation [13]. The effect of prolificacy on MY is likely larger in farms where kids are raised by their does. A similar practice is prevalent in the Transmontano ecotype of Serrana goat breed. The appearance of reproductive disorders, may interrupt prolactin stimulation of the udder, since it depends on the intensity of the suckling stimulus produced by different size litters, which may be the cause of the differences observed in mean MY. In spite of the negative effects of RD on MY, it is advantageous to have multiple fetuses in the herds. Prolificacy can be promoted using reproductive techniques [12]. Due to the inherent risk of RD when using these, any misclassification between abortion and dystocia should be avoided in order to improve benchmarking analysis of reproductive performance and of subsequent MY.

The interactions between the number of fetuses, parity, and RD on SMY150 observed in the present study suggest that the different management practices and environmental conditions can have a significant impact on MY and should be taken in consideration in herd health management. Transmontano ecotype of Serrana goats are normally reared for dual purpose and produce low MY under pastoralism. Hence, greater attention should be paid to this ecotype at a nutritional and milking (once vs. twice a day) level to improve the lactation following RD. 

In further studies, the exact moment of abortion should be considered mainly for primiparous goats, in which the mammary development occurs for the first time, in order to evaluate its influence on MY. 

## 5. Conclusions

We conclude that RD interacted with number of fetuses and parity in low-producing dairy goats modifying the SMY150.

A negative effect of RD on SMY150 was more pronounced for primiparous than multiparous goats and for goats carrying multiple fetuses than singletons, likely due to endogenous hormonal influence.

The goat lactation following abortion or stillbirths at kidding is viable under pastoralism and the improvement of production system management can play an important role in mitigating MY losses.

## Figures and Tables

**Table 1:** Effect of number of fetuses, parity, and reproductive disorders (RD), and their interactions, in Transmontano ecotype of Serrana goats 150-day standardized milk yield (SMY150).

Transmontano Ecotype of Serrana Goats				*p* Values	
Fetuses	Parity	Parturition	SMY150 (*n*)		
Single	Multiparous	Normal	97.5 ± 39.7 ^a^ (53,125)	No. of fetuses Parity RD	<0.05< 0.001< 0.001
		RD	93.9 ± 40.3 ^b^ (8278)		
	Primiparous	Normal	88.7 ± 32.7 ^c^ (21,048)	No. of fetuses × Parity No. of fetuses × RD Parity × RD	<0.001< 0.001 0.01
		RD	80.2 ± 31.5 ^d^ (4778)		
Multiple	Multiparous	Normal	100.9 ± 42.0 ^e^ (50,708)	No. of fetuses × Parity × RD	<0.05
		RD	86.7 ± 39.7 ^f^ (690)		
	Primiparous	Normal	98.3 ± 40.4 ^g^ (9321)		
		RD	79.8 ± 31.2 ^d,h^ (136)		

^a–h^ different superscript letters within the same column are significant at *p* < 0.05.

**Table 2:** Percentage variation of 150-day standardized milk yield in Transmontano ecotype of Serrana goats presenting reproductive disorders.

Transmontano Ecotype Of Serrana Goats ^(1)^		
Parity	Singleton	Multiple fetuses
Multiparous goats	−3.7%	−14.1%
Primiparous goats	−9.6%	−18.8%

^(1)^ Milk yield variation (%) = [SMY150 (RD) / SMY150 (normal) −1] * 100. SMY150 (RD): 150-day standardized milk yield from goats with reproductive disorders. SMY150 (normal): 150-day standardized milk yield from goats with normal parturition.
